# Diosgenin normalization of disrupted behavioral and central neurochemical activity after single prolonged stress

**DOI:** 10.3389/fphar.2023.1232088

**Published:** 2023-08-16

**Authors:** Hurmat Malik, Muhammad Usman, Mehreen Arif, Zainab Ahmed, Gowhar Ali, Khalid Rauf, Robert D. E. Sewell

**Affiliations:** ^1^ Department of Pharmacy, COMSATS University Islamabad, Abbottabad, Pakistan; ^2^ Department of Pharmacy, University of Peshawar, Peshawar, Pakistan; ^3^ Cardiff School of Pharmacy and Pharmaceutical Sciences, Cardiff University, Cardiff, United Kingdom

**Keywords:** single prolonged stress, diosgenin, post-traumatic stress disorder, behavioral test, neurochemical profiling

## Abstract

**Introduction:** Post-traumatic stress disorder (PTSD) is a chronic mental illness triggered by traumatic experiences such as wars, natural disasters, or catastrophes, and it is characterized by anxiety, depression and cognitive impairment. Diosgenin is a steroidal sapogenin with known neuroprotective and antioxidant properties. This study aimed to assess the pharmacological potential of diosgenin in a single prolonged stress (SPS) model of PTSD, plus other behavioral models along with any consequent alterations in brain neurochemistry in male mice.

**Methodology:** SPS was induced by restraining animals for 2 h, followed by 20 min of forced swim, recuperation for 15 min, and finally, exposure to ether to induce anesthesia. The SPS-exposed animals were treated with diosgenin (20, 40, and 60 mg/kg) and compared with the positive controls, fluoxetine or donepezil, then they were observed for any changes in anxiety/depression-like behaviors, and cognitive impairment. After behavioral screening, postmortem serotonin, noradrenaline, dopamine, vitamin C, adenosine and its metabolites inosine and hypoxanthine were quantified in the frontal cortex, hippocampus, and striatum by high-performance liquid chromatography. Additionally, animal serum was screened for changes in corticosterone levels.

**Results:** The results showed that diosgenin reversed anxiety- and depression-like behaviors, and ameliorated cognitive impairment in a dose-dependent manner. Additionally, diosgenin restored monoamine and vitamin C levels dose-dependently and modulated adenosine and its metabolites in the brain regions. Diosgenin also reinstated otherwise increased serum corticosterone levels in SPS mice.

**Conclusion:** The findings suggest that diosgenin may be a potential candidate for improving symptoms of PTSD.

## 1 Introduction

PTSD is a trauma-induced mental illness characterized by the intrusive reliving of past incidents, accompanied by negative thoughts, hyperarousal, avoidance, and terror (Enman et al., 2015; Lee et al., 2020). Concerning this, a distressing, potentially fatal incident can also result in the development of PTSD (Wilson et al., 2014). Symptoms associated with PTSD are dissociation, recurrent nightmares, recalls, intrusive thoughts, increased startle reaction, poor focus, interrupted sleep, and severely negative emotional states (Sherin and Nemeroff, 2022). Globally, the prevalence of PTSD among those who were exposed to trauma has been estimated to be present in 5.6% of the general population (White et al., 2023) while others have approximated it to be 9.2%–13.6% (Atwoli et al., 2015). During the COVID-19 pandemic, the overall pooled estimated incidence rate of PTSD from a total of 24 countries was as high as 17.52% (Yunitri et al., 2022).

PTSD involves structural and neurochemical abnormalities in different brain regions together with increased oxidative stress. The neurochemical abnormalities chiefly involve catecholamine imbalances in the neuroendocrine system (Cohen et al., 2009), and the amygdala, prefrontal cortex, and hippocampus are key brain areas associated with PTSD pathophysiology (Quinones et al., 2020). The frontal cortex is responsible for executive function and fear extinction, while the medial prefrontal cortex controls stress responses and the regulation of emotion via inhibitory control in association with its amygdaloidal connections. The prefrontal cortex also influences striatal brain activity to regulate habitual, orientated, and goal-directed behavior (Falconer et al., 2008; Piggott et al., 2019). Moreover, in models of PTSD, there is evidence of an adverse effect not only on hippocampal volume (Felmingham et al., 2009) but there are also reduced serotonin levels coupled with elevated noradrenaline concentrations reflecting an enhanced noradrenergic response (Wilson et al., 2014).

The striatum regulates goal-directed, motivated, and habitual behaviors that are disrupted in PTSD due to loss of inhibitory control of the pre-frontal cortex over the striatum (Piggott et al., 2019; Sambuco et al., 2021). Striatal monoamines and vitamin C were quantified because their levels are disrupted in the striatum during PTSD. Hence, serotonin modulates dopamine levels in the striatum (Krystal and Neumeister, 2009) which has a defined role in pre-frontal cortical-associated working memory (Landau et al., 2009; D’Ardenne et al., 2012).

In addition, aberrant serotonergic transmission disrupts the equilibrium between the amygdala and hippocampus, which increases angiogenesis (Lee et al., 2020). Others have shown that noradrenaline and dopamine levels are increased, and serotonin is reduced in the hippocampus, prefrontal cortex, and amygdala, which are key structures in the fear response and the regulation of emotions such as anxiety via hypothalamic stimulation (Krystal and Neumeister, 2009; Hoexter et al., 2012; Krishnamurthy et al., 2013). Furthermore, hypothalamic-pituitary-adrenocortical (HPA) dysregulation, oxidative stress, and monoamine (dopamine serotonin, and noradrenaline) imbalances are contributory elements in a PTSD model (Lee et al., 2020). Despite ongoing stress, patients with PTSD have elevated corticotrophin-releasing factor (CRF) and normal to decreased cortisol levels (Katrinli et al., 2022b). In this context, CRF and cortisol control noradrenaline release from the locus coeruleus resulting in an increased sympathetic tone and hallmark symptoms of PTSD, such as a heightened startle response and hyperarousal (DUNN et al., 2004; Seki et al., 2018).

Vitamin C is an aqueous-soluble vitamin with both antioxidant and neuroprotective properties. It has been shown to reduce memory impairment in animal models of PTSD, in addition to influencing catecholamines and corticosterone, these actions are thought to be derived from the prevention of oxidative stress in the brain (Alzoubi et al., 2020).

Adenosine is a neuromodulator and the adenosinergic system can be neuroprotective as well as neurodegenerative depending on how it is differentially manipulated, for example, by A_1_ receptor activation, A_2A_ receptor antagonism or inhibition of adenosine kinase (Gomes et al., 2011). What is more, adenosine levels markedly increase during brain damage and this occurs most probably because of increased ATP consumption to maintain cell viability (Latini and Pedata, 2001; Gomes et al., 2011). In conjunction with this, adenosine and its metabolites have an established role in mood regulation and cognitive impairment (Elmenhorst et al., 2018; van Calker et al., 2019).

Both psychotherapy and pharmacotherapy are employed in the management and treatment of PTSD. Regarding pharmacotherapy, the FDA-approved drugs for PTSD as a first-line therapy, are the serotonin selective reuptake inhibitors (SSRIs). An α_1_-adrenoceptor blocker, prazocin, has also been used off-label for trauma nightmares (Miller, 2008). However, a high relapse rate (>60 %) is associated with these drug therapies (Wahbeh et al., 2016; Akiki and Abdallah, 2019) and they tend to be used with caution, especially in older patients with lower tolerability (Draper and Berman, 2008). There is, therefore, a need for the development of novel agents with improved efficacy and limited toxicity for the management and treatment of PTSD.

Diosgenin is a hydrolysate of dioscin and can be extracted from Dioscorea plant tubers. Dioscin is a natural saponin, while diosgenin is a saponin derivative (Li et al., 2021). These two compounds are extensively present in Liliaceae, Dioscoreaceae, Leguminosae, Solanaceae, and Agavaceae plant families (Avula et al., 2014; Yuan et al., 2019; Semwal et al., 2022) with proven anti-atherosclerotic (Wang and Wang, 2022) and anti-asthmatic activities (Junchao et al., 2017). Both Diosgenin and Dioscin reduce oxidative stress by inhibiting ROS production and increasing superoxide dismutase expression (Qin et al., 2013). Dioscin also reduces RAGE and NOX4 expression yielding an anti-Alzheimer’s disease effect (Guan et al., 2022). Additionally, it has antidepressant-like activity and is neuroprotective, reducing inflammation by decreasing IL-6, IL-1β, and TNF-α levels (Yang et al., 2017; Qi et al., 2019).

The neuroprotective profile of diosgenin confers effectiveness against diabetes-induced neuropathy (Leng et al., 2020) and cerebral ischemic brain injury (Oyelaja-Akinsipo et al., 2020), whilst also possessing antioxidant and anti-inflammatory capacities (Yang et al., 2020) plus an ability to improve cognitive deficits in senescent mice (Chiu et al., 2011; Semwal et al., 2022). More recently, diosgenin has been shown to have a pharmacological propensity against chronic restraint stress-induced depression (Cui et al., 2023). Despite its clear neuroprotective and anti-inflammatory properties, the pharmacological potential of diosgenin has not been explored in a murine model of PTSD and this was the primary aim of this study using SPS in combination with short-term memory and anxiety/depression-like models.

## 2 Materials and methods

### 2.1 Animals

Male BALB/c mice, 26–30 g were used in the experimental study, and they were obtained from the animal house facility of COMSATS University Islamabad, Abbottabad Campus. Mice were kept in a humidity-temperature-controlled room on a 12–12 h light-dark cycle. All animals were provided with food and water *ad libitum*. The Ethical Care Committee at the COMSATS University Islamabad, Abbottabad campus approved all the experimental protocols under the approved letter number PHM **.** Eth/CS-M01-019-2901.

### 2.2 Materials

Diosgenin (CAS number 512-04-9, purity ≥98%) acetonitrile, serotonin, citrate buffer, dopamine adenosine, and the Corticosterone Competitive ELISA kit (Invitrogen, catalog number EICORT) were procured from Sigma Aldrich while fluoxetine and donepezil were purchased from Aries Pharma, Peshawar.

### 2.3 Experimental protocol

Six mice were allocated to each group with a total of seven groups. Group 1, was not subjected to any stress but administered normal saline; Group 2, was subjected to 7-day SPS and received intraperitoneal (i.p.) daily normal saline (i.e., control); Group 3, following SPS, animals received daily fluoxetine (10 mg/kg, i.p., i.e., anxiolytic/antidepressant positive control); Group 4, following SPS, was treated with daily donepezil (4 mg/kg i.p. i.e., nootropic positive control); Groups 5, 6, and 7, following SPS, were treated with diosgenin at doses of 20, 40 or 60 mg/kg i.p. respectively and selected from earlier studies (Kiasalari et al., 2017; Cui et al., 2023).

#### 2.3.1 Induction of the single prolonged stress (SPS) model

**FIGURE 1:**
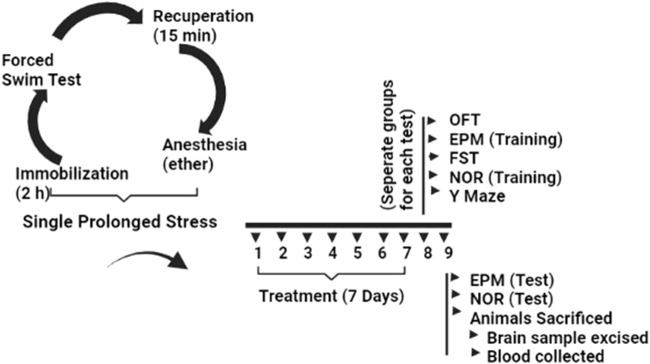
Experimental protocol for induction of PTSD via SPS and evaluation of behavioral and neurochemical changes after diosgenin treatment.

All experimental mice were systematically subjected to stress throughout three sessions in the SPS model. Mice were first immobilized for 2 hours in restrain tubes, after which they were immediately forced to swim for 20 min in glass tanks (10 cm × 25 cm), filled with water at a level of 15 cm (temperature 24°C). The mice were recuperated for 15 min and then exposed to ether vapor until they lost consciousness. Finally, the mice were kept in their home cages without being interrupted for 7 days to develop symptoms of PTSD. After 7 days, certain behavioral tests were performed to screen various parameters. Animals used as controls were not stressed in any way (Liberzon et al., 1999; Yang et al., 2022), (Figure 1).

### 2.4 Behavioral evaluation

Experiments were performed to evaluate various parameters of PTSD-like behavior. All the behavioral tests were performed between 9:00 a.m. and 2:00 p.m. To avoid accumulative stress, separate groups of animals were used for evaluating each behavioral test.

#### 2.4.1 Elevated plus maze (EPM)

Animals were observed in the EPM to evaluate anxiety-like behavior. The apparatus was suspended 70 cm from the floor and had four arms in total, two of which were open and two of which were closed, each measuring 10 cm in width and 45 cm in length. The closed arms had 10 cm high walls, but there were no walls on the open arms (Handley and Mithani, 1984) Individual mice were gently positioned in the central area (5 × 5 cm) of the compartment, facing the open arm, at the beginning of each test. The maze was carefully cleaned between each trial with 70% alcohol to avoid any smell signature, and a video camera was used to record animal arm location timings for a total of 5 min. The frequency of entry and the time spent in open versus closed arms during occupancy were assessed. These parameters were used to derive the anxiety index using the following formula (Carola et al., 2002; Yoshizaki et al., 2020):

Anxiety Index = 1 − [([Open arm time/test duration] + [Open arm entries/total number of entries])/2].

#### 2.4.2 Open field test (OFT)

The OFT measured spontaneous locomotor activity using activity boxes with uniform dimensions (46 × 46 cm) and a floor divided into four quadrants (23 × 23 cm). Individual mice were introduced to the laboratory 4 hours before each trial, and they were habituated to the apparatus for 20 min. Individual mice were placed in the center of the box and a video camera was set above the arena to record locomotor activity for 30 min (Arif et al., 2022). In assessing spontaneous locomotion and anxiety-like behavior, the number of lines crossed (30 min), rearing and incorrect transitions in grooming bouts were recorded for 6 min duration (Kalueff et al., 2004; Denmark et al., 2010).

#### 2.4.2 Forced swim test (FST)

The FST was performed following a modified method of (Porsolt et al., 1977). Individual mice were placed in a water tank (10 cm × 25 cm, with a water level of 15 cm and a temperature maintained at 25.0°C ± 1.0°C) and were forced to swim for 6 min. The time (s) for which mice remained motionless was recorded following 1 min of habituation. When an animal stopped attempting to swim and was only able to maintain its head above water, it was deemed to be immobile.

#### 2.4.3 Novel object recognition (NOR)

An open field box with a blackened surface (50 × 25 × 50 cm) was used as the arena for the NOR test. Mice were familiarized with the test box two consecutive days before the trial to avoid the effect of anxiety and stress on the results. Individual animals were introduced to an empty box on the first day (also known as habituation day), and they were permitted to explore for 10 min without the presence of objects. Mice were then placed separately in the box on the following day (training day) and allowed for 10 min to interact with two comparable objects that were kept apart from one another. After 24 h (test day), each animal was exposed to a familiar object and a novel object of a similar size (Hendrickx et al., 2022) and the time spent with either object was recorded by video camera (Ennaceur and Aggleton, 1997). When an animal’s head was facing the object, or when the distance between its head and the object was 1 cm or less, or when the animal was sniffing or touching the object, it was regarded to be exploration time (Garofalo et al., 2023). Between each experiment, 70% ethanol was used to clean the items and the box (Arif et al., 2022). A recognition index (RI) and a discrimination index (DI) were derived from the data (Hendrickx et al., 2022) as follows:

$$RI=\frac{Time\;\left(new\right)\times 100}{Time\;\left(new\right)+Time\left(old\right)}$$

$$DI=\frac{Time\;\left(new\right)-Time\;\left(old\right)}{T\left(total\right)}$$

#### 2.4.4 Y-maze

Spatial memory was assessed using a Y-maze apparatus, which was comprised of three arms of equal length positioned at 120°, each arm measuring 21 cm, length × 40 cm, height ×8.5 cm, width. Individual mice were placed in the center to explore the maze freely for 5 min. A video camera was used for recording animal activity in the maze. The parameters recorded for analysis included total arm entries, number of alternations, percentage alternations, and the number of triads. Arm entries were documented whenever the hind paws were present in any given arm, and sequential full entry into each arm has deemed an alternation. The percentage alternations were calculated using the formula (Prieur and Jadavji, 2019).

% alternations = (total number of alternations) × 100/Number of arms entered

### 2.5 Quantification of serum corticosterone

Following behavioral tests, animals were decapitated, and trunk blood was collected and allowed to coagulate for 30 min. The blood was then centrifuged for 20 min at 15,000 rpm at 4°C (Yakhkeshi et al., 2022). The serum was separated and kept at −80°C until it was analyzed. Serum corticosterone levels were measured using a Corticosterone Competitive Elisa kit (Invitrogen, catalog number EICORT, assay range 78.125–10,000 pg/mL, analytical 234 sensitivity of 18.6 pg/mL) following the manufacturer’s instructions. To summarize, repeated dilutions of reference standards were prepared to establish a standard curve. A 5 µL volume of serum was mixed with 55 µL of dissociation reagent to make serum samples and 490 µL of assay buffer was added to each sample after vortex mixing and incubating the sample for 5 min to prepare a 1:100 dilution. Standard concentrations from 5000 pg/mL to 78 pg/mL, as well as unknown samples, were added to respective wells. All the samples were run in duplicate. Then, 25 µL of corticosterone conjugate and corticosterone antibody were added to each well and the plate was shaken horizontally for 1 h at room temperature in an orbital shaker. Tetramethylbenzidine substrate (TMB) was then added, and after 30 min, a stop solution (1M HCl) was added, and the color change was evaluated within 10 min using an ELISA reader (Multiskan™ FC Microplate Photometer) at 450 nm. The standard curve was generated using a four-parameter algorithm. Background absorbance was subtracted from all data points and concentration was multiplied by the dilution factor.

### 2.6 Quantification of neurotransmitters and their metabolites

After decapitation, brains were excised, and the striatum, frontal cortex and hippocampus were dissected on an ice-cold plate and preserved in Eppendorf tubes at −80°C to prevent neurotransmitter degradation. After homogenizing the isolated brain areas in 0.2 percent perchloric acid, samples were homogenized at 5,000 rpm using a Teflon glass homogenizer (Ultra-Turax®T-50), then samples were centrifuged at 4°C and 120,000 rpm for 20 min (DLAB Scientific). The supernatant was filtered through a 0.45 mm filter (CNW technologies) before being injected into an HPLC autosampler for analysis (Sottofattori et al., 2001; Malik et al., 2023).

#### 2.6.1 Chromatographic conditions

HPLC analysis was performed using a Waters Alliance 2690 separation module equipped with an auto-sampler, UV detector, and PDA (United States). A C18 column (250 × 4.6 mm, 5 µm particle size) (Waters X Select® HSS Ireland) was used, and the mobile phase employed for quantification of noradrenaline, serotonin, dopamine and vitamin C was composed of 20 mM monobasic sodium phosphate and methanol (95:5, v/v), the detection is performed at 280 nm, at a column temperature of 35°C and flow rate of 0.5 mL/min (Tokhi et al., 2023). The mobile phase used for quantification of adenosine, inosine, and hypoxanthine was composed of acetonitrile (5:95, v/v) and 0.01M monobasic sodium phosphate. The flow rate was 0.01 mL/min, at a column temperature of 35°C, and detection was performed at 260 nm using isocratic elution in phosphate buffer (Ur Rehman et al., 2020).

#### 2.6.2 Calibration curve and standard preparation

The calibration curve was constructed using several dilutions of noradrenaline, serotonin, dopamine, vitamin C, adenosine, hypoxanthine, and inosine. Each standard stock solution was prepared at a concentration of 1.0 mg/10 mL and from this, dilutions of 100, 200, 300, 400, and 500 ng/mL were prepared. The samples were loaded into an auto-sampler, and a 20 µL sample was stipulated in the software for injection (Empower TM). Calibration curve was created using linear regression analysis by graphing the peak area (y) against the concentration (x). By comparing the respective peak areas, unknown concentrations of neurotransmitters, vitamin C, adenosine and its metabolites, were determined (Hou et al., 2019; Tokhi et al., 2023; Usman et al., 2023).

### 2.7 Statistics

Graph Pad prism was used to analyze the data and the mean ± SEM was expressed for each group (n = 6). A normality test was performed on all data sets using the Shapiro-Wilk test. One way ANOVA was applied with *post hoc* Dunnett’s test for analysis and *p* < 0.05 was adopted as the threshold for significance.

## 3 Results

### 3.1 Effect of diosgenin or fluoxetine on modified anxiety-like behavior induced by SPS in the elevated plus maze (EPM)

**FIGURE 2:**
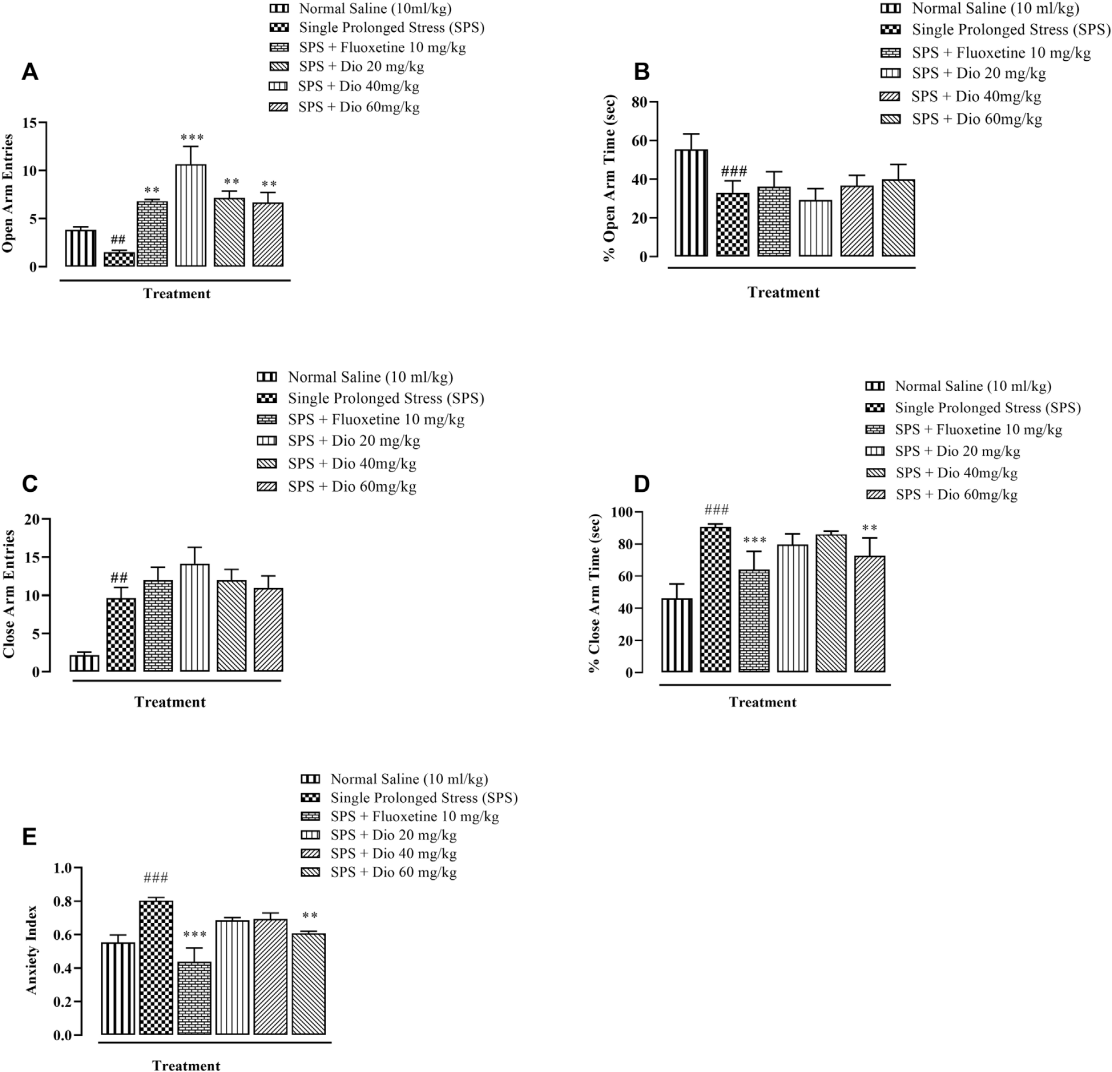
Effect of diosgenin (Dio) or fluoxetine treatment (i.p.) on mouse behavior in the EPM (elevated plus maze test of 5 min duration) in animals exposed to SPS. The figure presents the incidence of open arm entries **(A)**, % open arm occupancy time **(B)**, incidence of closed arm entries **(C)**, % closed arm occupancy time **(D)** and anxiety index **(E)**. ^*###*^ *p* < 0.001, versus the saline group. *p < 0.05, ***p < 0.001 versus the SPS group.

In the EPM, open-arm entries were significantly decreased in the SPS-exposed animal group compared with saline-treated mice, and this SPS-induced decrease was completely reversed by both diosgenin and fluoxetine as a positive control (Figure 2A, F (5, 30) = 11.44, *p* < 0.0001). There was also a reduction in the percentage of open-arm occupancy time induced by SPS which was not modified by either diosgenin or fluoxetine (Figure 2B, F (5, 30) = 18.02, *p* < 0.0001). Concomitantly, there was a marked elevation of closed-arm entries generated by SPS which remained unchanged by fluoxetine or diosgenin (Figure 2C F (4, 25) = 5.978, *p* = 0.0016). However, the percentage of closed-arm occupancy time was also raised by SPS, but it was reversed by fluoxetine and the highest dose of diosgenin (Figure 2D F (5, 30) = 24.90, *p* < 0.0001). The anxiety index was derived from the open arm occupancy times and entries, as well as the overall total number of entries to the test duration. Subsequent calculation of the anxiety index revealed that it was augmented by exposure to SPS and it was then reversed by fluoxetine and diosgenin (60 mg/kg) (Figure 2E, F (5, 30) = 9.081 *p* < 0.0001).

### 3.2 Effect of diosgenin or fluoxetine on modified behaviors in the open field test (OFT) induced by SPS

**FIGURE 3:**
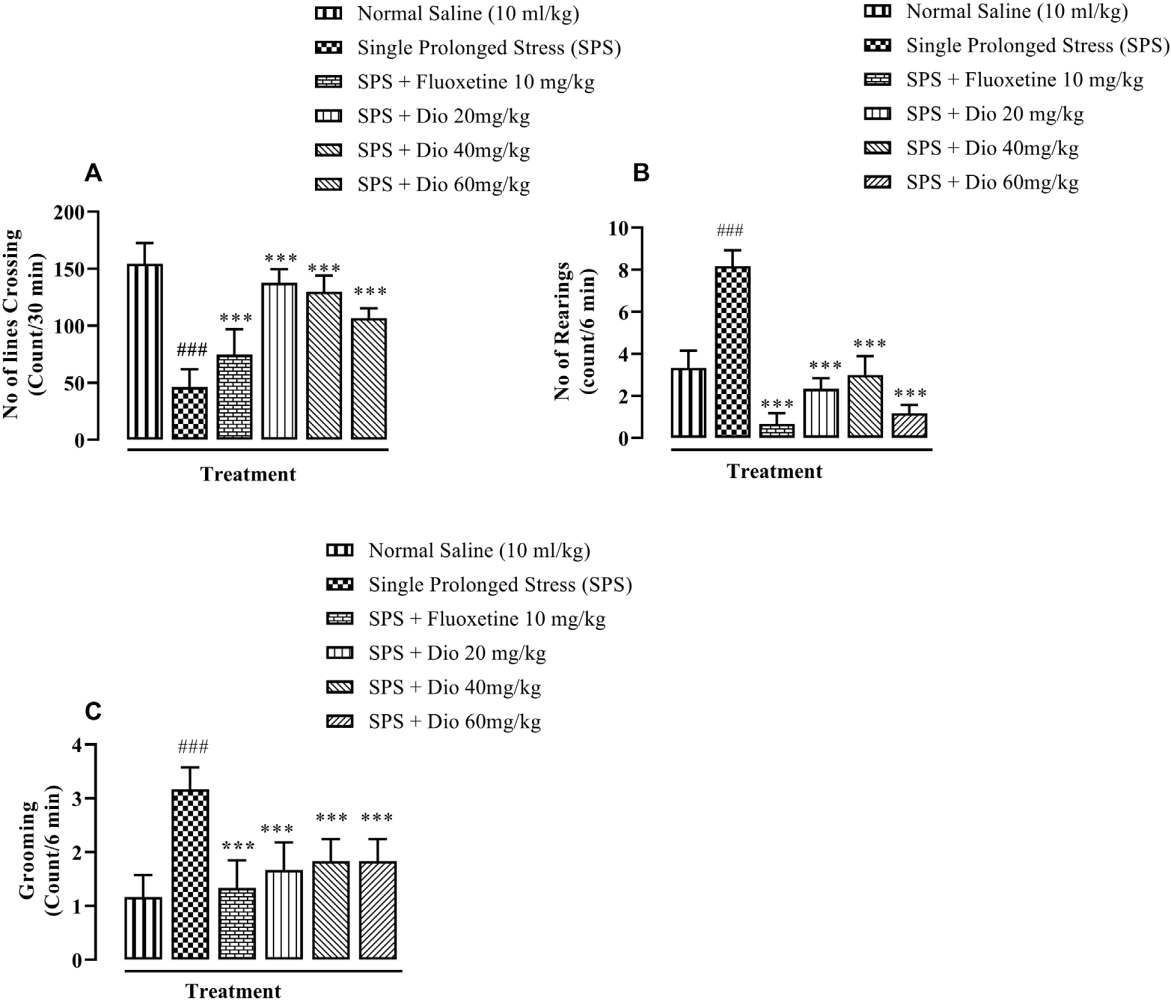
The effect of diosgenin (Dio) or fluoxetine treatment (i.p.) on SPS-modified behavioral activity of mice in the open field test (OFT). The figure presents spontaneous locomotor activity **(A)**, rearing behavior **(B)** and the number of grooming episodes increased by SPS **(C)**. ^###^ *p* < 0.001, versus the saline control group. *p < 0.05, ***p < 0.001, versus the SPS exposed group.

In OFT, there was a sizeable reduction in the rate of line crossings in mice exposed to SPS. Fluoxetine (positive compared to the SPS group. Thus, both compounds reversed the SPS suppression of locomotor activity and in the case of the lowest diosgenin dose, the outcome activity was comparable to that expressed by the saline control group. (Figures 3A, F (5, 30) = 40, *p* < 0.0001). In the SPS-exposed animals, there was a marked rise in rearing behavior which was reversed by fluoxetine and diosgenin at all 3 doses (Figures 3B, F (5, 30) = 94.78, *p* < 0.0001). Likewise, the incidence of grooming behavior was also significantly increased in the SPS-exposed group, and then it was reduced by either fluoxetine or diosgenin to levels analogous to vehicle controls (Figures 3B, F (5, 30) = 22.57 *p* < 0.0001).

### 3.3 Effect of diosgenin or fluoxetine on modified behavioral despair in the forced swim test induced by SPS

**FIGURE 4:**
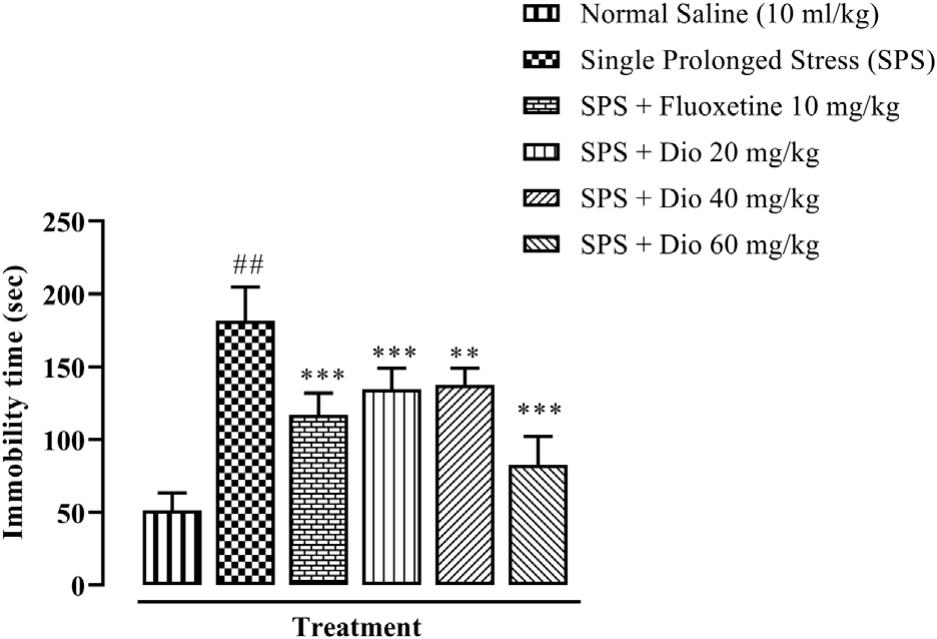
Effect of fluoxetine or diosgenin (Dio) treatment (i.p.) on immobility time in the forced swim test (FST) in mice exposed to SPS. ^##^ *p* < 0.05, versus the saline control group. **p < 0.01, ***p < 0.001, versus the SPS-exposed group.

There was a substantial protraction of immobility time in mice exposed to SPS, as an expression of behavioral despair in FST. Both the positive control, fluoxetine, and the three doses of diosgenin reversed the SPS extended immobility time to levels approaching those of saline vehicle-treated control animals (Figure 4, F (5, 30) = 46.03, *p* < 0.0001).

### 3.4 Effect of diosgenin or donepezil on novel object recognition (NOR) memory induced by SPS

**FIGURE 5:**
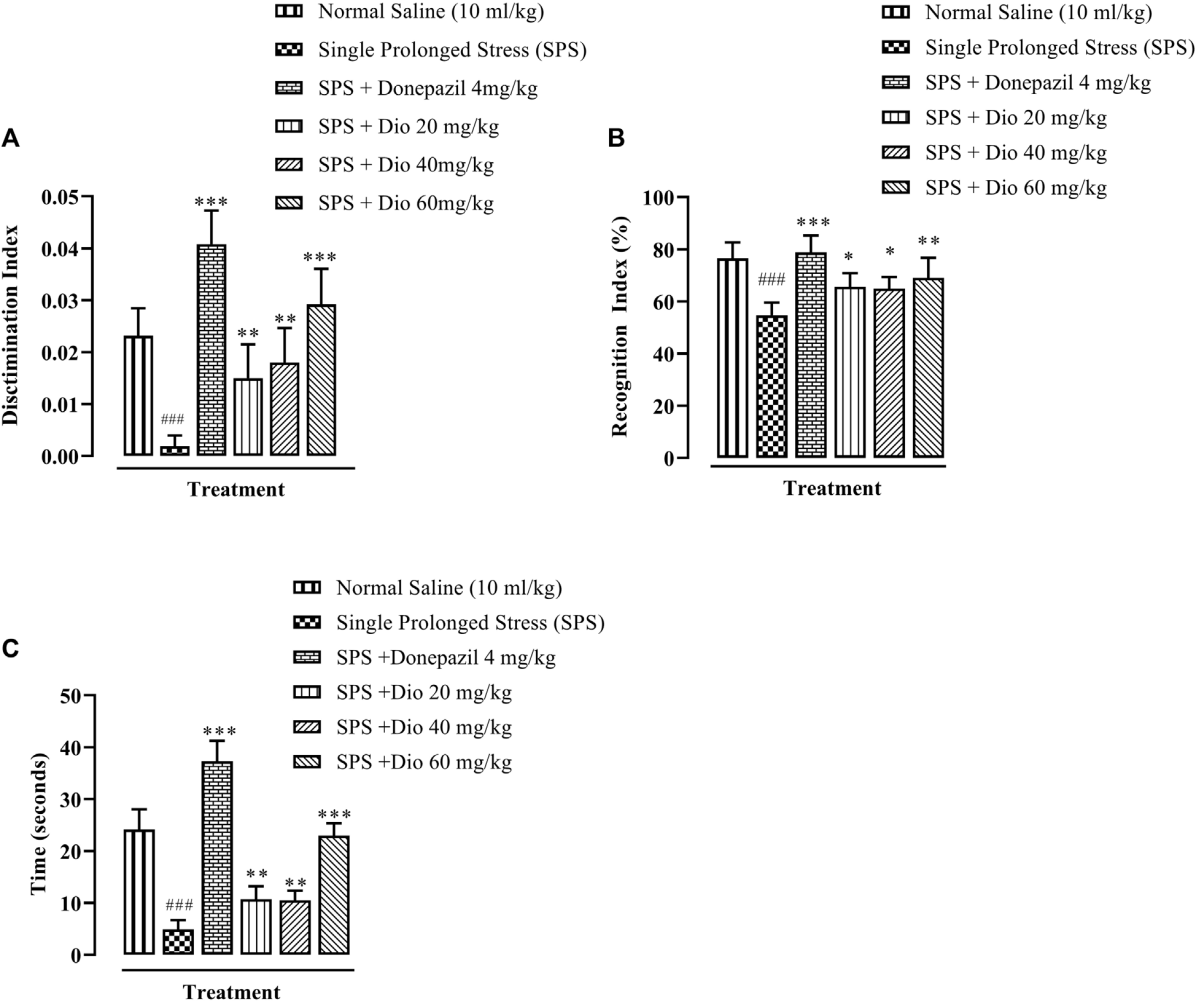
Effect of donepezil or diosgenin (Dio) treatment (i.p.) on impaired cognition in the novel object recognition (NOR) test (over a 10 min trial duration) in mice exposed to SPS. The figure presents the discrimination index (DI) of a novel object versus a familiar object **(A)**, recognition index (RI) expressed in terms of percentage **(B)** and the time of exploration engaged with a novel object (seconds) **(C)**. ^###^ *p* < 0.001 versus the saline control group. *p < 0.05, ^**^ *p* < 0.01, ^***^ *p* < 0.001 versus the SPS exposed group.

In the NOR paradigm, there was a significant decrease in the discrimination index (DI) in mice exposed to SPS. Diosgenin at all 3 doses, as well as the positive control, donepezil, significantly reversed the SPS-impaired DI (Figure 5A, F (5, 29) = 30.30 *p* < 0.0001). Similarly, there was a deterioration in the recognition index (RI) evoked by SPS exposure, which was subsequently reversed by donepezil and diosgenin (Figure 5B, F (5, 24) = 10.98, *p* < 0.0001). Correspondingly, the time spent with the novel object was also substantially reduced in mice exposed to the SPS protocol, while donepezil and diosgenin both caused a prolongation of the SPS shortened time engaged with the novel object (Figure 5C, F (5, 30) = 105.1, *p* < 0.0001).

### 3.5 Effect of diosgenin or donepezil on modified Y-Maze behavior induced by SPS

**FIGURE 6:**
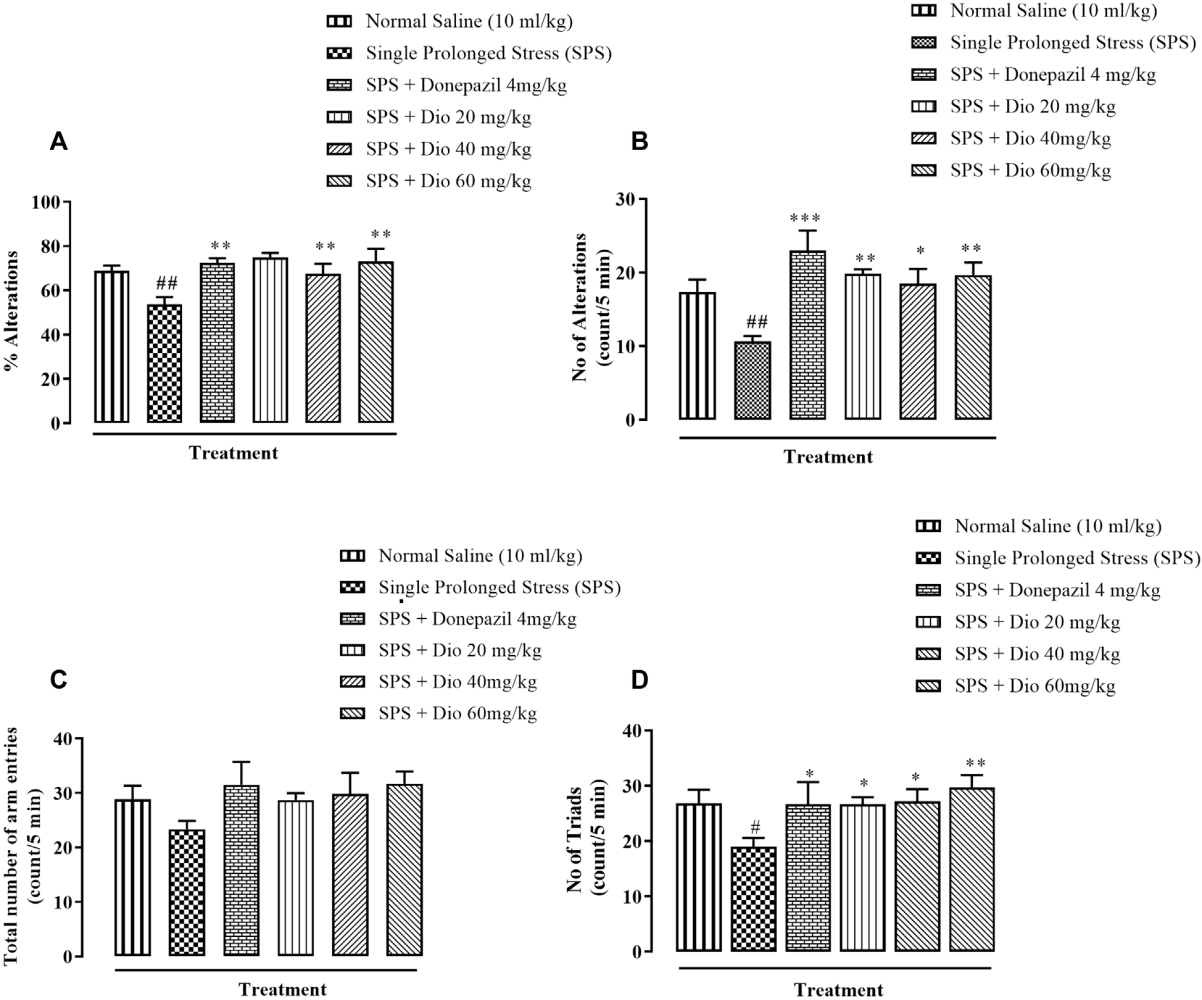
Effect of diosgenin (Dio) or donepezil treatment (i.p.) on mouse behavior in the Y-maze paradigm conducted in mice exposed to SPS. The figure presents animal percentage alternations **(A)**, number of alternations **(B)**, the total number of animal entries **(C)**, and number of triads **(D)**. ^#^ *p* < 0.05, ^##^ *p* < 0.01 versus the saline control group. *p < 0.05, **p < 0.01, ***p < 0.001: versus the SPS exposed group.

There was a significant reduction in percentage alternations in the Y-maze task displayed by mice that underwent the SPS protocol in comparison with the saline vehicle-treated control group. However, donepezil (positive control) and the two higher doses of diosgenin significantly increased the percentage alternations in comparison with the SPS group (Figure 6A, F (4, 25) = 5.086, *p* = 0.0039), while the SPS reduced number of alternations were reversed by donepezil and all doses of diosgenin (Figure 6B, F (4, 25) = 5.086, *p* = 0.0039) There were no differences observed in the total number of entries across all treatment groups (Figure 6C, F (5, 30) = 1.161, *p* = 0.3509) although exposure to SPS suppressed the number of triads which were subsequently reinstated by donepezil or diosgenin administration (Figure 6D, F (4, 25) = 2.658, *p* = 0.0564).

### 3.6 Effect of diosgenin or fluoxetine on elevated serum corticosterone concentration induced by SPS

**FIGURE 7:**
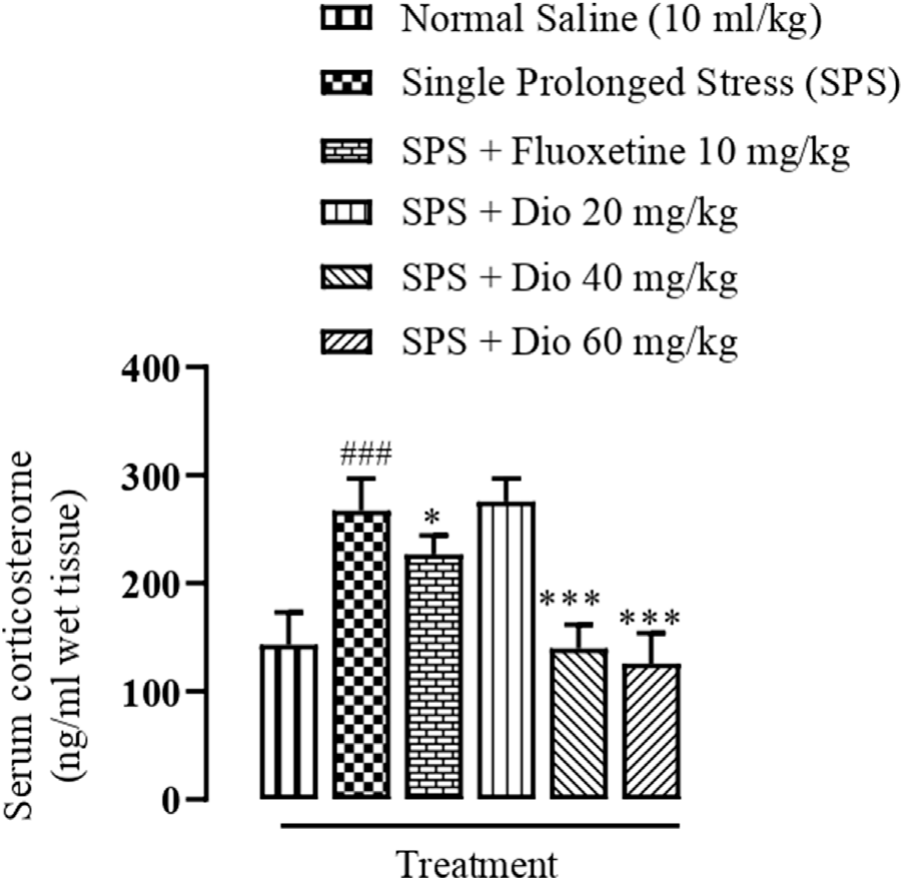
Effect of diosgenin (Dio) or fluoxetine treatment (i.p.) on raised serum levels of corticosterone induced by the SPS protocol in mice. ^###^ *p* < 0.001 versus the saline control group. ^*^ *p* < 0.05, ^***^ *p* < 0.001 versus the SPS exposed group.

The serum corticosterone concentration was significantly raised above saline-treated control levels in mice exposed to the SPS protocol. In contrast, the SPS elevated serum corticosterone was brought down by fluoxetine and diosgenin 40 mg/kg and 60 mg/kg), and the levels were normalized to that equivalent to controls (Figure 7, F (5, 30) = 44.19, *p* < 0.0001**).**

### 3.7 Effect of diosgenin, donepezil, or fluoxetine on modified frontal cortical concentrations of noradrenaline, dopamine, serotonin, and vitamin C induced by SPS

In SPS-exposed mice, noradrenaline and dopamine levels were significantly increased in the frontal cortex, however, no changes were observed in serotonin, though vitamin C levels were decreased in the SPS-exposed group. In contrast, diosgenin reduced noradrenaline levels at the highest doses, F (5, 30) = 18.63, *p* < 0.0001, F (5, 30) = 26.87, *p* < 0.0001 though it did not produce any significant effects on dopamine, and only fluoxetine and donepezil were able to lower the levels of dopamine. It was notable that diosgenin at the highest test dose (60 mg/kg) elevated the SPS-suppressed concentration of vitamin C, F (5, 30) = 6.109, *p* = 0.0005 (Table 1).

**TABLE 1:** Effect of diosgenin (Dio), fluoxetine, or donepezil treatment (i.p.) on frontal cortical alterations in noradrenaline, dopamine serotonin and vitamin C (ng/mg of wet tissue) after exposure to SPS. ^##^ *p* < 0.01, ^###^ *p* < 0.001 versus the saline control group. ^*^ *p* < 0.05, ***p < 0.001 versus the SPS-exposed group.

Treatments	Noradrenaline	Dopamine	Serotonin	Vitamin C
Normal Saline (10 ml/kg)	46.3 ± 2.2	22.2 ± 1.9	27.7 ± 0.6	19.7 ± 1.5
Single Prolonged Stress (SPS)	71.5 ± 1.7^##^	46.5 ± 3.2^###^	26.7 ± 2.7	13.9 ± 1.6^##^
SPS + Dio 20 mg/kg	57.2 ± 3.8*	42.1 ± 1.3	21.78 ± 1.4	16.5 ± 1.3
SPS + Dio 40 mg/kg	44.6 ± 3.8***	41.4 ± 3.1	12.3 ± 1.0	15.4 ± 1.2
SPS + Dio 60 mg/kg	43.8 ± 1.9***	38.9 ± 3.6	10.5 ± 1.5	22.3 ± 1.3***
SPS + Donepezil 4 mg/kg	43.7 ± 1.0***	9.9 ± 2.2***	30.35 ± 5.6	17.9 ± 1.4
SPS + Fluoxetine 10 mg/kg	70.8 ± 2.0	27.8 ± 1.0***	37.3 ± 1.3	19.9 ± 0.6

### 3.8 Effect of diosgenin, donepezil, or fluoxetine on modified hippocampal concentrations of noradrenaline, dopamine, serotonin, and vitamin C induced by SPS

In SPS-exposed mice, noradrenaline and dopamine levels in the hippocampus were significantly increased in the SPS-exposed group. This effect was reversed by diosgenin, fluoxetine, and donepezil F (6, 31) = 28.86, *p* < 0.0001, F (5, 30) = 26.87, *p* < 0.0001. In contrast, serotonin and vitamin C levels were diminished in the SPS-exposed group. Subsequently, the concentrations of serotonin were increased by fluoxetine, donepezil, and diosgenin F (5, 27) = 26.49, *p* < 0.0001, but vitamin C was raised only by the highest dose of diosgenin (Table 2).

**TABLE 2:** Effect of diosgenin (Dio), fluoxetine or donepezil treatment (i.p.) on hippocampal changes in noradrenaline, dopamine serotonin and vitamin C (ng/mg of wet tissue) after exposure to SPS. ^###^ *p* < 0.001 versus the saline group, ^*^ *p* < 0.01, ^**^ *p* < 0.01, ^***^ *p* < 0.001 versus the SPS group.

Treatments	Noradrenaline	Dopamine	Serotonin	Vitamin C
Normal Saline (10 ml/kg)	16.8 ± 1.6	7.2 ± 1.3	14.3 ± 1.9	5.1 ± 0.6
Single Prolonged Stress	25.2 ± 2.4 ^###^	46.1 ± 2.0^###^	1.2 ± 0.1^##^	2.0 ± 0.1^###^
SPS + Dio 20 mg/kg	19.0 ± 0.8*	17.9 ± 2.9 ***	13.9 ± 1.4***	1.4 ± 0.1
SPS + Dio 40 mg/kg	10.0 ± 0.7***	23.3 ± 2.6***	6.7 ± 0.3**	1.9 ± 0.2
SPS + Dio 60 mg/kg	17.1 ± 0.9***	21.9 ± 0.5***	7.5 ± 0.9***	3.0 ± 0.1*
SPS + Donepezil 4 mg/kg	11.5 ± 1.5***	5.6 ± 0.6***	5.6 ± 0.6*	3.0 ± 0.2*
SPS + Fluoxetine 10 mg/kg	21.0 ± 1.6	28.9 ± 2.0**	7.8 ± 0.6***	2.1 ± 0.1

### 3.9 Effect of diosgenin, donepezil, or fluoxetine on modified striatal concentrations of noradrenaline, dopamine, serotonin, and vitamin C induced by SPS

In SPS-exposed mice, noradrenaline, and dopamine levels in the striatum were significantly increased and then reversed by donepezil and diosgenin at all three doses, F (6, 35) = 175.9, *p* < 0.0001, F (6, 35) = 40.39, *p* < 0.0001. In contrast, serotonin, and vitamin C levels were diminished in the SPS-exposed group, and subsequently, serotonin levels were increased by fluoxetine, and diosgenin at the highest, F (5, 28) = 19.56, *p* < 0.0001, while vitamin C was increased by donepezil and diosgenin at all doses, F (6, 33) = 17.74, *p* < 0.0001 (Table 3).

**TABLE 3:** Effect of diosgenin (Dio), fluoxetine, or donepezil treatment (i.p.) on striatal changes in noradrenaline, dopamine, serotonin and vitamin C (ng/mg of wet tissue) after exposure to SPS. ^###^ *p* < 0.001 versus the saline group. ^*^ *p* < 0.05, ^**^ *p* < 0.01, ^***^ *p* < 0.001 versus the SPS group.

Treatments	Noradrenaline	Dopamine	Serotonin	Vitamin C
Normal Saline (10 ml/kg)	48.6 ± 2.4	12.7 ± 2.1	21.7 ± 1.7	4.5 ± 0.6
Single Prolonged Stress (SPS)	73.8 ± 3.9 ^###^	24.2 ± 1.4^###^	0.6 ± 0.3^###^	1.7 ± 0.1^###^
SPS + Dio 20 mg/kg	5.1 ± 0.5***	14.0 ± 1.6^***^	3.0 ± 0.3	4.3 ±0.3^**^
SPS + Dio 40 mg/kg	4.9 ± 0.8***	17.4 ± 2.0^***^	2.6 ± 0.3	6.1 ± 0.7^***^
SPS + Dio 60 mg/kg	4.9 ± 0.2***	6.4 ± 1.1^***^	3.2 ± 0.2*	8.2 ± 0.5^***^
SPS + Donepezil 4 mg/kg	8.8 ± 2.1 ^***^	3.9 ± 1.2^***^	3.6 ± 1.3**	3.8 ± 0.4^*^
SPS + Fluoxetine 10 mg/kg	65.1 ± 2.6	27.9 ± 2.6	10.5 ± 7.4***	2.8 ± 0.1

### 3.10 Effect of diosgenin, donepezil, or fluoxetine on modified frontal cortical concentrations of adenosine, inosine, and hypoxanthine induced by SPS

In SPS-exposed mice, adenosine and inosine levels were increased in the frontal cortex and then significantly reduced by donepezil, fluoxetine, and all 3 doses of diosgenin F (6, 28) = 117.5, *p* < 0.0001, F (6, 28) = 77.50, *p* < 0.0001. However, no significant differences were observed in hypoxanthine levels after SPS exposure or after diosgenin, donepezil, or fluoxetine treatment F (6, 35) = 4.080, *p* = 0.0033 (Table 4).

**TABLE 4:** Effect of treatment with diosgenin (Dio), fluoxetine, or donepezil (i.p.) on changes in adenosine, inosine, and hypoxanthine concentrations (ng/mg of wet tissue) in the frontal cortex after SPS. ^###^ *p* < 0.001 versus the saline group. **p < 0.01, ***p < 0.001 versus the SPS group.

Treatments	Adenosine	Inosine	Hypoxanthine
Normal Saline (10 ml/kg)	5.0 ± 0.3	4.6 ± 0.3	7.0 ± 0.3
Single Prolonged Stress (SPS)	13.4 ± 0.6^###^	51.8 ± 3.0^###^	6.5 ± 0.3
SPS + Dio 20 mg/kg	5.6 ± 0.5**	38.4 ± 2.1***	5.3 ± 0.2
SPS + Dio 40 mg/kg	1.9 ± 0.2***	20.3 ± 2.8***	6.5 ± 0.4
SPS + Dio 60 mg/kg	1.0 ± 0.1***	15.6 ± 1.3***	5.0 ± 0.2
SPS + Donepezil 4 mg/kg	2.3 ± 0.1***	45.2 ± 2.3	6.4 ± 0.2
SPS + Fluoxetine 10 mg/kg	4.6 ± 0.3**	11.2 ± 0.5***	5.3 ± 0.4

### 3.11 Effect of diosgenin, donepezil, or fluoxetine on modified hippocampal concentrations of adenosine, inosine, and hypoxanthine induced by SPS

In SPS-exposed mice, inosine levels were elevated in the hippocampus and then significantly reduced by donepezil, fluoxetine, and all 3 doses of diosgenin F (6, 34) = 49.47, *p* < 0.0001. However, no significant differences were observed in adenosine F (6, 28) = 1.528, *p* = 0.2056, and hypoxanthine levels after the SPS protocol, F (6, 33) = 0.4005, *p* = 0.8733 (Table 5).

**TABLE 5:** Effect of treatment with diosgenin (Dio), fluoxetine, or donepezil (i.p.) on changes in adenosine, inosine, and hypoxanthine concentrations (ng/mg of wet tissue) in the hippocampus after SPS. ^*###*^ *p* < 0.001 versus the saline group, ***p < 0.001 versus the SPS group.

Treatments	Adenosine	Inosine	Hypoxanthine
Normal Saline (10 ml/kg)	2.8 ± 0.2	2.7 ± 0.4	16.2 ± 1.3
Single Prolonged Stress (SPS)	3.0 ± 0.2	9.2 ± 0.5^###^	18.2 ± 1.2
SPS+ Dio 20 mg/kg	2.9 ± 0.3	3.2 ± 0.4***	16.7 ± 1.3
SPS + Dio 40 mg/kg	3.2 ± 0.3	5.6 ± 0.6***	17.3 ± 0.3
SPS + Dio 60 mg/kg	3.1 ± 0.2	2.8 ± 0.3***	15.8 ± 0.2
SPS+ Donepezil 4 mg/kg	2.5 ± 0.1	8.9 ± 0.5	18.2 ± 1.6
SPS + Fluoxetine 10 mg/kg	3.0 ± 0.2	0.4 ± 0.9***	17.2 ± 2.5

### 3.12 Effect of diosgenin, donepezil, or fluoxetine on modified striatal concentrations of adenosine, inosine, and hypoxanthine induced by SPS

In SPS-exposed mice, the concentration of adenosine was significantly increased in the striatum but subsequently reduced by fluoxetine, and diosgenin at higher doses, F (6, 31) = 12.97, *p* < 0.0001. However, no significant alterations were observed in inosine F (6, 30) = 1.172, *p* = 0.3471, and hypoxanthine levels after the SPS protocol F (5, 25) = 1.778, *p* = 0.1539 (Table 6).

**TABLE 6:** Effect of treatment with diosgenin (Dio), fluoxetine, or donepezil on changes in adenosine, inosine, and hypoxanthine concentration (ng/mg of wet tissue) in the striatum after SPS exposure. ###*p* < 0.001 versus the saline group, *p < 0.01, **p < 0.01, ***p < 0.001 versus the SPS group.

Treatments	Adenosine	Inosine	Hypoxanthine
Normal Saline (10 ml/kg)	3.6 ± 0.3	4.0 ± 0.2	4.2 ± 0.3
Single Prolonged Stress (SPS)	5.1 ± 0.3^##^	3.8 ± 0.1	3.9 ± 0.3
SPS + Dio 20 mg/kg	4.9 ± 0.2	3.3 ± 0.1	4.3 ± 0.2
SPS + Dios 40 mg/kg	3.4 ± 0.1**	3.8 ± 0.2	4.4 ± 0.3
SPS + Dio 60 mg/kg	3.1 ± 0.1***	3.9 ± 0.1	3.5 ± 0.19
SPS + Donepezil 4 mg/kg	3.4 ± 0.2**	3.8 ± 0.2	3.9 ± 0.1
SPS + Fluoxetien 10 mg/kg	2.3 ± 0.2**	3.9 ± 0.1	3.0 ± 0.2

## 4 Discussion

We have evaluated the effect of treatment with diosgenin in the SPS rodent model of PTSD. Male mice were used in our experiments in congruence with other studies (Valdivieso et al., 2018; Lee et al., 2020), because they are more vulnerable than females, who have higher basal corticosterone levels, and male mice also react to stress less adaptably than females (Cohen and Yehuda, 2011). This particular model was employed because it produces stress-like responses arising from different source mechanisms, such as restraint for psychological stress, forced swimming for physiological stress, and ether to induce pharmacological stress (Lisieski et al., 2018). The protocol is intended to simulate the major corticosterone surge caused by the experience of a traumatic event (Armario et al., 2008). SPS is one of the most frequently used paradigms for evoking PTSD-related symptoms in rodents, including depression, anxiety, and impaired cognition (Souza et al., 2017).

In recent studies, mice exposed to SPS have been shown to display a higher anxiety index in the EPM paradigm (Figure 2), as well as decreased locomotor activity, incorrect transitions in grooming bouts, and increased rearing in the OFT (Figure 3), reflecting anxious behavior, increased immobility time in FST suggests that the animals are more fearful together with learned helplessness after experiencing trauma (Figure 4) and our findings are in accord with previous studies (Lee et al., 2018b; Lin et al., 2022; Sur and Lee, 2022), mirroring the anxiety and behavioral despair in PTSD patients. Diosgenin reduced the anxiety index (Figure 2E), the number of open arm entries (Figure 2A), diminished the percentage closed arm occupancy time at the highest dose (Figure 2D). These results are analogous to those obtained with the positive control, fluoxetine, where there was a correlation with lowered serotonin levels in the frontal cortex, hippocampus, and striatum, together with an increased anxiety index (Lee et al., 2020). Reduced serotonin levels in response to stress may be accompanied by aggression and mood changes, such as regret, depression, and anxiety since serotonin is centrally engaged in emotional and behavioral regulation, which influences aggressive behavior (Lee et al., 2020; Fluyau et al., 2022). In our study, diosgenin restored the otherwise SPS-reduced levels of serotonin in the hippocampus (Table 2), with very little effect on striatal serotonin (Table 3), conceivably signifying an anxiolytic-like effect also seen with fluoxetine. It is well documented that in rodent models fluoxetine is used as a positive control in the murine model of PTSD as it ameliorates the anxiety and depression-like symptoms associated with PTSD (Lee et al., 2018a; Oh et al., 2018; Lee et al., 2020). Moreover, fluoxetine after acute treatment has been reported to ameliorate the anxiety and depression-like behavior in PTSD (Oh et al., 2018) and chronic unpredictable stress (Ahmed et al., 2023). Diosgenin, like fluoxetine, not only increased locomotor activity (Figure 3A) but also reduced the incidence of rearing (Figure 3B) and incorrect transitions in grooming (Figure 3C). Excessive grooming or incorrect transitions in grooming patterns and rearing behaviors reflect anxiogenesis (Kruk et al., 1998; Kalueff et al., 2004; Nagarajan and Capecchi, 2023). These are typical rodent responses to stressful situations involving dysregulation of the HPA axis (Moody et al., 1988; Kinlein et al., 2019) resulting in high corticosterone levels, which ultimately give rise to abnormal stress processing (Patki et al., 2014). We observed that the serum corticosterone level was raised following SPS exposure, which was then reversed by diosgenin (Figure 7), and the degree of restoration even surpassed that of fluoxetine as the standard positive control. In humans, decreased peripheral cortisol levels are associated with PTSD, but these changes do not appear in the acute stage ensuing traumatic stress (Shalev et al., 2008; Videlock et al., 2008). In our study, plasma corticosterone levels were assessed during the initial stages after stress exposure, so there is no discord between our findings and those reporting that PTSD patients have lower cortisol levels (Mongeau et al., 1997; Morgese and Trabace, 2019). Diosgenin has reported antidepressant-like effect by decreasing serum pro-inflammatory cytokines and reducing the HPA axis activity (Cui et al., 2023). We have shown that diosgenin and fluoxetine both attenuated SPS elevated serum corticosterone to reverse HPA axis dysregulation (Figure 7). In addition, increased CRF or cortisol levels in response to stress or aversive stimuli results in noradrenaline release from locus coeruleus (LC) neurons (Yehuda et al., 1991; DUNN et al., 2004) leading to increased sympathetic tone and hallmark symptoms of PTSD, such as an increased startle response and hyperarousal (DUNN et al., 2004; Seki et al., 2018), and increased release of dopamine in the striatum, In addition to HPA axis dysregulation, a close relationship exists between monoamine dysfunction and PTSD-associated depression. Most antidepressants modulate the levels of both noradrenaline and serotonin in a time-dependent manner (Porsolt et al., 1977; Geracioti Jr et al., 2001) and these monoamines regulate each other through heteroreceptors. Negative feedback has been proposed because elevated serotonin levels cause noradrenaline production, which in turn blocks further serotonin release by activating α2A receptors (Mongeau et al., 1997). Hence, serotonergic receptors at noradrenergic terminals and α2A receptors (inhibitory in nature) on serotonergic terminals mediate this mechanism (Mongeau et al., 1997; Morgese and Trabace, 2019). Restoration of the HPA axis and monoamines in these brain regions resulted in the amelioration of SPS-induced anxiety and depression-like symptoms. Moreover, endogenous vitamin C levels were disrupted by SPS in the frontal cortex (Table 1), hippocampus (Table 2), and striatum (Table 3), and they were restored by diosgenin in these brain areas. Vitamin C affects signal transduction in the brain via modulation of neurotransmitter release and receptor binding (Hansen et al., 2014). During the synthesis of monoamines, Vitamin C serves as a co-factor (Rebec and Pierce, 1994; Kocot et al., 2017) and regulates dopamine and noradrenaline levels (May et al., 2012; Moritz et al., 2020). Vitamin C supplementation is associated with the protection of serotonin from oxidation, resulting in its increased uptake, receptor binding and turnover, with a likelihood of ameliorating anxiety/depression-like symptoms (Ballaz and Rebec, 2019). Reduced vitamin C levels in the brain are associated with a reduction in brain serotonin levels (Ward et al., 2013). SPS disrupts vitamin C levels in different brain regions, subsequently altering monoamine concentrations in the brain.

During neuropathological conditions, central adenosine levels are abruptly increased, exacerbating the ongoing pathology (van Calker et al., 2019). Adenosine is an additional neuromodulator involved in the pathophysiology of depression. It also plays a critical role in other biological processes, including the regulation of mood and behavior, by acting on various receptors such as the A_1_, A_2A_, A_2B_, and A_3A_ subtypes (Pasquini et al., 2022). It is reported that adenosine and its analogs produced depressant-like behavioral effects in animal models of depression (Woodson et al., 1998; Hunter et al., 2003; Cunha et al., 2008). Thus, elevated adenosine levels extended the immobilization time in rats subjected to inescapable shocks as well as in the FST. Adenosine is converted to inosine in the presence of adenosine deaminase, which is further converted to hypoxanthine in the presence of purine nucleoside phosphorylase, then to xanthine, and finally to uric acid in the presence of xanthine oxidase. Inosine is formed inside the cell by adenosine deamination when the level of intracellular adenosine is high (Haskó et al., 2004). Activation of A_3_ receptors by inosine has an additional anti-inflammatory effect (Haskó et al., 2004) and inosine also has neuroprotective activity (Saad et al., 2022). However, when inosine and hypoxanthine levels are abnormally high, they are ultimately metabolized to uric acid, which is responsible for the generation of hydrogen peroxide and ROS leading to neurodegeneration (Frenguelli and Dale, 2020). We observed a comparable outcome since the adenosine level was increased by SPS and subsequently reversed by diosgenin in the frontal cortex (Table 4) and striatum (Table 6). Additionally, inosine levels in the frontal cortex (Table 4) and hippocampus (Table 5) were boosted by SPS and then attenuated by diosgenin and fluoxetine concurring with the immobility time findings in the FST (Figure 3). However, no significant change was observed in hypoxanthine levels, which may be ascribed to the rapid conversion of hypoxanthine to xanthine in the presence of xanthine oxidase (Frenguelli and Dale, 2020).

Furthermore, Adenosine A_2A_ receptor activation modulates memory and learning through the presynaptic release of glutamate, and these adenosinergic receptor sub-types are also thought to play a role in memory and learning by facilitating synaptic plasticity (Chen, 2014). Increased adenosine and inosine levels in the frontal cortex following SPS exposure were then reduced by diosgenin, and this may well play a part in improving memory in the NOR task.

PTSD causes cognitive impairment, coupled with reduced recognition (Keller et al., 2015) and an impairment of spatial memory (Schoenfeld et al., 2019). Recognition memory is the capacity to identify something familiar when previously observed (Baxter, 2010) and it is used to evaluate rodent object recognition memory in NOR which if impaired, may reflect amnesia in humans (Baxter, 2010). NOR memory was reduced in the SPS-exposed animal group in our study, and this concurs with an earlier finding (Eagle et al., 2013). In this regard, it has been shown that damage to the frontal cortex and hippocampus, which are crucial to object recognition and spatial memory, can cause anterograde amnesia. Hence, diosgenin and donepezil both increased the DI (Figure 5A) and RI (Figure 5B) values degraded by SPS. Moreover, the reduction in memory impairment by diosgenin was comparable to that of donepezil, as the positive control. Donepezil is an FDA-approved drug clinically used to address cognitive problems. It is reported to augment fear extinction in PTSD and enhance memory, and has a neuroprotective role (Dong et al., 2009; Lim et al., 2016; Reid et al., 2018; Gomaa et al., 2021; Yanpallewar et al., 2022). In this respect, donepezil serves as a positive control in murine models of cognitive impairment (Arif et al., 2022).

It is well known that dopaminergic function is vital for the functioning of working memory. Thus, the dopamine concentration in the amygdala increases and activation of dopamine receptors is crucial for the storage of memories associated with fear. Dopamine concentrations in the striatum and prefrontal cortex mediate cognition and executive functioning, including attentiveness and the inhibitory response (Ding et al., 2023). In this context, overstimulation of the dopamine system in animal models is linked to both the strengthening of fear conditioning and delayed extinction of fear (Morrow et al., 1996). Moreover, high dopamine levels are associated with intensified fear conditioning responses to cues that resemble “trauma,” compromising the capacity to ignore unimportant stimuli and assign proper salience (Hoexter et al., 2012). Furthermore, neuronal serotonin transmission in the hippocampus plays a pivotal role in memory processing via long-term potentiation (LTP) and depression (LTD.) most likely involving 5-HT7 receptors (Sarkisyan and Hedlund, 2009). Diosgenin may exert its effect on NOR by modulating serotonin, particularly since it increased its concentration in the hippocampus and striatum in our study. Likewise, noradrenaline levels were modified in the frontal cortex, hippocampus, and striatum. In this regard, noradrenaline has an established role in memory formation, consolidation, and recovery (Hamidkhaniha et al., 2019). Additionally, dopamine levels were also increased in the frontal cortex, hippocampus, and striatum, which is in accord with previous studies; (Baxter, 2010; Wilson et al., 2014). In addition, donepezil decreased SPS disrupted dopamine levels in all three brain regions (Tables 1–3) suggesting that restoration of monoamines in distinct brain regions may be involved in the amelioration of SPS-induced cognitive impairment in the NOR test.

The Y-maze protocol is used for the assessment of spatial working memory by evaluating spontaneous alternations that reflect the functional interplay between the frontal cortex and hippocampus. We found a reduction in spontaneous alternations following the SPS protocol, which reflects negative cognition, and corroborates the findings of others (Fulco et al., 2022). Donepezil and diosgenin both increased the SPS reduction in spontaneous alternations (Figure 6A), improving the short-term working memory affected by SPS.

A convincing association between the pathophysiology of PTSD and oxidative stress has been reported in animal studies (Wilson et al., 2013; Miller et al., 2018; Li et al., 2020; De Munter et al., 2021) and human trials (Katrinli et al., 2022). The disrupted HPA axis in PTSD may also be involved in oxidative stress because high levels of glucocorticoids in response to excitotoxicity may result in an increased production of proinflammatory cytokines. Increased oxidative stress is responsible for neuronal damage in the frontal cortex and hippocampus (MacPherson et al., 2005), which is likely to impair learning. Vitamin C has an antioxidant potential that helps to oppose neurodegeneration in several ailments by conserving the antioxidant mechanism in the brain, imparting neuroprotection (Dwita et al., 2023), and ameliorating memory by restoring antioxidant mechanisms in the hippocampus. Through oxidation, vitamin C is hypothesized to shield cellular DNA, lipids, and proteins from oxidative damage caused by ROS and this can improve cognition (Alqudah et al., 2018). Additionally, conserving vitamin C levels have been associated with improved symptoms of PTSD **(** Cuthrell, 2022 **)**. In light of this, an added issue concerning the study of vitamin C in our study is that it is a co-factor in the metabolism of monoamines (Hansen et al., 2014), so it has an interrelationship and impact on the range of neurotransmitters that have been measured.

Our observation that vitamin C levels were diminished in all three brain regions after SPS, probably contributed to memory impairment due to perturbed antioxidant mechanisms. This can be correlated with preclinical findings suggesting that deficient endogenous vitamin C levels result in oxidative stress and neurodegeneration (Ballaz and Rebec, 2019). Subsequently, both donepezil and diosgenin raised the otherwise SPS-reduced levels of vitamin C in the frontal cortex (Table 1) and striatum (Table 3) conceivably participating in improved memory in the NOR and Y maze tasks.

PTSD is known to be a comorbidity with depression, anxiety, and alcohol abuse, so its underlying mechanisms of action are complex and diverse (Cai et al., 2022). The disorder involves disruption of the HPA axis, inflammation, as well as modified neurotransmission, and neurotropic function (Aliev et al., 2020; Cai et al., 2022). This, diosgenin has neuroprotective and antioxidant activity, modulates neurotransmitters, is anti-apoptotic and anti-inflammatory, attenuates Ca^2+^ influx modulating neurotrophic factors, inhibits tau phosphorylation, and can regenerate neural networks (Sun et al., 2015). Some of these activities may well stem from the repression of TNF-α, IL-1β, and IL-6 levels and the NF-κB pathway (Zhang et al., 2017; Man et al., 2023) in addition to the aforementioned actions. Because of these diverse properties, diosgenin can generate mnemonic/antidepressant/anxiolytic outcomes in our behavioral paradigms following SPS exposure.

Accordingly, it may be postulated that it has conferred neuroprotection during PTSD induced by single prolonged stress through one or more of its recognized mechanisms. It might be proposed consequently, that diosgenin would be useful not only in the treatment of PTSD but also in the management of PTSD-related ailments.

## 5 Conclusion

In summary, our findings suggest that diosgenin has anxiolytic- and antidepressant-like effects in addition to enhancing memory in the NOR and Y-maze tasks after the SPS protocol. Furthermore, amelioration of these post-SPS behavioral parameters may be attributed to the normalization of monoamines and vitamin C in the frontal cortex, hippocampus, and striatum, as well as the reinstatement of plasma corticosterone levels and other actions. Such outcomes suggest that diosgenin may be a potential candidate for improving symptoms of PTSD.

### 5.1 Limitations of the study

This study involves certain behavioral features of PTSD and concomitant neurochemical changes only, so further insight at the molecular level is warranted to explore the mechanism of Diosgenin in PTSD.

## Data Availability

The original contributions presented in the study are included in the article/Supplementary Material, further inquiries can be directed to the corresponding author.
